# Unconventional Secretion, Gate to Homeoprotein Intercellular Transfer

**DOI:** 10.3389/fcell.2022.926421

**Published:** 2022-06-28

**Authors:** Alain Joliot, Alain Prochiantz

**Affiliations:** ^1^ INSERM U932, Institut Curie Centre de Recherche, PSL Research University, Paris, France; ^2^ Centre for Interdisciplinary Research in Biology (CIRB), Collège de France, CNRS UMR 7241, INSERM U1050, PSL Research University, Labex MemoLife, Paris, France

**Keywords:** PIP 2, internalization, intercelluar communication, paracrine action, homeoprotein, unconventional secretion

## Abstract

Unconventional secretion allows for the secretion of fully mature and biologically active proteins mostly present in the cytoplasm or nucleus. Besides extra vesicle-driven secretion, non-extravesicular pathways also exist that specifically rely on the ability of the secreted proteins to translocate directly across the plasma membrane. This is the case for several homeoproteins, a family of over 300 transcription factors characterized by the structure of their DNA-binding homeodomain. The latter highly conserved homeodomain is necessary and sufficient for secretion, a process that requires PI(4,5)P2 binding, as is the case for FGF2 and HIV Tat unconventional secretion. An important feature of homeoproteins is their ability to cross membranes in both directions and thus to transfer between cells. This confers to homeoproteins their paracrine activity, an essential facet of their physiological functions.

## Introduction

Unconventional protein secretion gathers multiple and heterogenous pathways defined by absence of the hallmarks that characterize the conventional pathway, such as the presence of a signal sequence at the N-terminus of the secreted polypeptide and the use of an invariant endoplasmic reticulum to Golgi journey blocked by Brefeldin A (Viotti, 2016). Beside relying on alternative routes to cross the plasma membrane, unconventional secretion is uncoupled from translation and therefore, can concern fully mature proteins endowed with genuine intracellular functions. Extracellular vesicle-driven secretion, described in this issue, proved to be predominant for unconventional secretion pathways but it necessarily requires the entrapment of the secreted proteins within vesicles. In parallel, a limited set of proteins devoid of signal sequence were shown to accumulate freely in the culture medium. Among them, the growth factor FGF2 is one of the first reported example, and the mechanism of its secretion was accurately dissected (Steringer and Nickel, 2018). Homeoprotein secretion was first described 20 years ago (Joliot et al., 1998). This observation was unexpected as this protein family was originally identified as a class of transcriptional regulators. Importantly, homeoproteins are not only secreted but also internalized by cells, the combination of these two processes allowing their transfer between cells. Such transfer confers to homeoproteins a paracrine mode of action, now recognized as an essential component of their developmental and physiological functions.

## The Homeoprotein Family

Homeoproteins were discovered in a genetic screen focused on development in *Drosophila melanogaster* (Shearn et al., 1971). Some of the genes identified, named homeotic due to their ability to control the spatial identity of metameric structures, shared a common DNA-binding motif called the homeodomain (Gehring et al., 1994). Homeodomain-containing proteins, or homeoproteins, constitute one of the largest family of transcription factors highly conserved during evolution. More than 300 members are found in the human, where they exert multiple functions throughout life, as *bona fide* transcriptional regulators (Holland et al., 2007).

Originally, it is in the course of experiments aiming at perturbing the transcriptional activity of homeoproteins by mechanical loading of a purified homeodomain fragment in fragilized neuronal cells, we made the unexpected observation of homeodomain spontaneous uptake (Joliot A. et al., 1991). Later on, we demonstrated that full-length homeoproteins are efficiently internalized and also secreted, despite the absence of a classical secretion signal sequence (Joliot et al., 1998). Once in the extracellular medium, homeoproteins are detected in the soluble fraction following 100,000×*g* centrifugation (Joliot et al., 1998) and are able to interact with cell surface carbohydrates (Layalle et al., 2011), ruling out their incorporation into extracellular vesicles. This behavior is similar to that described for FGF2 and the HIV Tat protein, distinct to homeoproteins although similarly highly basic, suggesting the possibility of similarities in secretion mechanisms. It was then demonstrated that these unusual trafficking properties confer to homeoproteins new functions that superimpose on their transcriptional activity. They will be specifically discussed in the last part of this review.

## Homeoprotein Secretion

**FIGURE 1:**
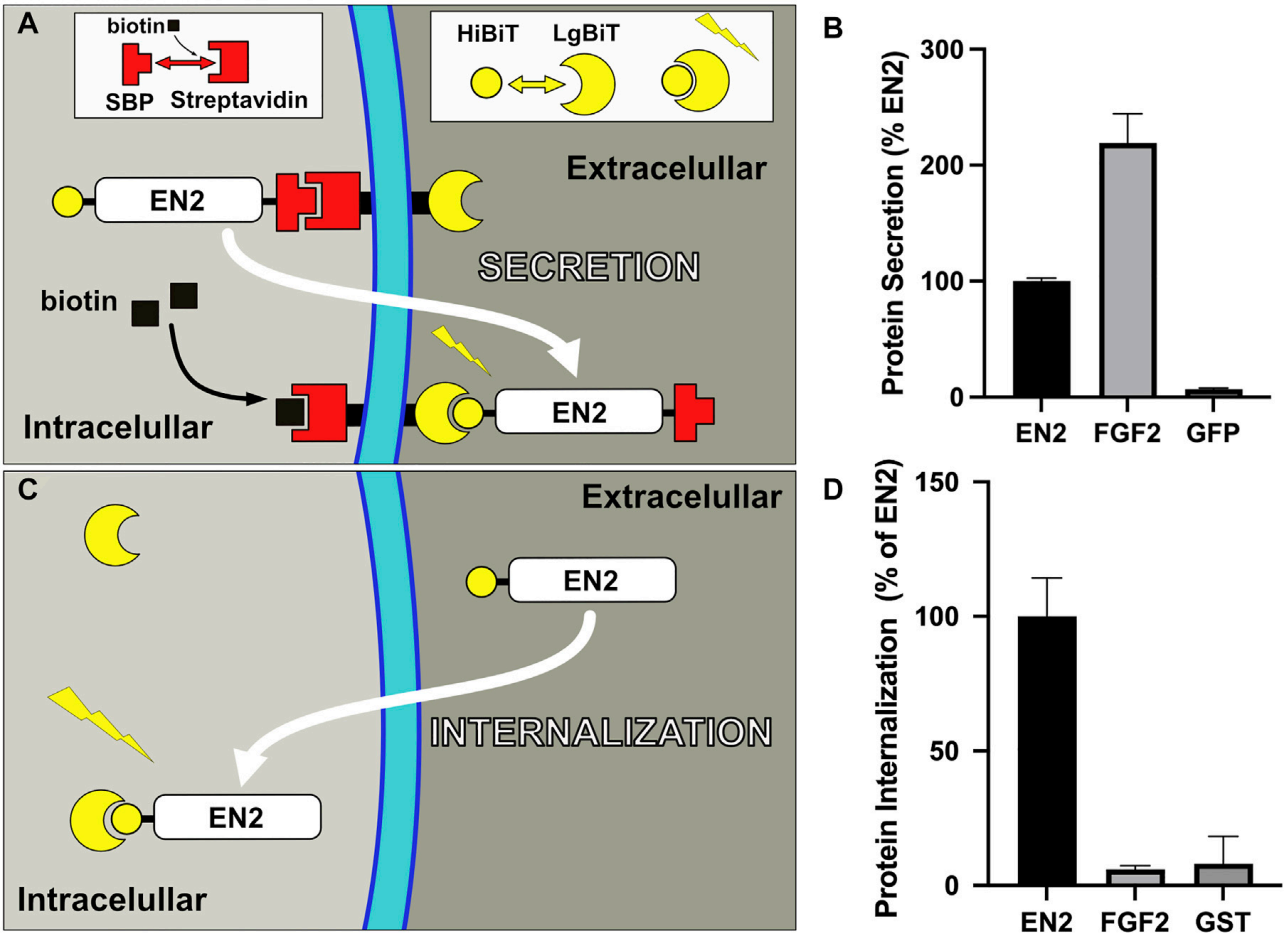
Quantitative translocation assays. **(A)** Secretion assay (TransRush): Thanks to the addition of a SBP tag, EN2 is hooked at the inner side of the plasma membrane using the Rush system and can be released upon biotin addition. The presence of a second tag (HiBiT) allows monitoring secretion of EN2 upon release through complementation with the complementary Nanoluc fragment (LgBiT) present at the cell surface. **(B)** Quantification of the secretion of the indicated proteins with the TransRush assay. Luciferase activity is quantified 1 h after biotin addition. **(C)** Internalization assay: Cytosolic delivery of a HiBiT-tagged recombinant EN2 protein loaded in the medium is monitored through complementation with the complementary Nanoluc fragment (LgBiT) present in the cytosol of the recipient cell. **(D)** Cytosolic delivery of the indicated proteins is quantified 30 min after addition in the medium (**B**,**D** from ref 22).

Homeoproteins predominantly localize to the nucleus, as expected for transcription factors. By subcellular fractionation, they are also detected in the membrane fraction and selectively distribute into raft domains that are characteristic of the plasma membrane (Joliot et al., 1997). Since it is estimated that no more than 10% of the intracellular pool of homeoproteins is secreted (Maizel et al., 1999), a sensitive assay is required to monitor their secretion. In a recent study, we have implemented a new strategy called TransRush, combining the Ru system to control protein trafficking (Boncompain et al., 2012) and nanoluciferase bi-molecular complementation (Dixon et al., 2016) to monitor secretion (Figure1A). Thanks to the addition of two tags, the protein is hooked at the inner side of the plasma membrane using the RUSH system, and its accumulation in the extracellular space monitored through bi-molecular complementation with a luciferase fragment attached at the outer side of the plasma membrane. Secretion of the hooked protein is quantified following its release by biotin addition compared to control conditions and normalized by the cell content (Figure 1B). Both FGF2 and chick Engrailed2 homeoprotein (EN2) secretions could be accurately quantified, revealing a higher secretion efficacy for the former (Amblard et al., 2020a).

The mechanism of homeoprotein secretion was precisely dissected with EN2, used as a paradigm for this protein family. It shows striking similarities with that of FGF2 (Temmerman et al., 2008) and HIV Tat (Rayne et al., 2010) proteins and in particular, a mandatory requirement for Phosphatidylinositol(4,5)bisphosphate [PI(4,5)P2] (Amblard et al., 2020a). PI(4,5)P2 are minor components of the cell lipidome but specifically localize in the inner leaflet of the plasma membrane (Borges-Araújo and Fernandes, 2020). The efficacy of EN2 secretion strictly correlates with the levels of PI(4,5)P2, modulated by enzymatic or pharmacologic treatments. PI(4,5)P2 are known to be essential for the recruitment of various proteins at the cytosolic face of the plasma membrane, such as proteins involved in actin remodeling and in signal transduction (McLaughlin et al., 2002). They act in a similar way with FGF2, Tat and EN2, allowing for their recruitment at their site of secretion. Indeed, EN2 directly interacts with PI(4,5)P2 in artificial bilayers (Amblard et al., 2020a). The nature of the lipid polar head is an important determinant of EN2 interaction as PI4P and PS, show decreasing affinity for EN2. Comparing the respective affinities of the three proteins for PI(4,5)P2 is uneasy due to the diversity of techniques used but when analyzed with a same setting, FGF2 and EN2 display similar affinities (Amblard et al., 2020a). In a live cell context, EN2 interaction with PI(4,5)P2 is supported by the delocalization of the PI(4,5)P2-sensor PHPLC ∂ upon induction of EN2 secretion and by the release of EN2 from membranes treated with neomycin, a classical PI(4,5)P2 competitor.

Contrasting with FGF2, EN2 significantly interacts with PI4P, with a fourfold lower affinity for this lipid compared to PI(4,5)P2, but PI4P could not substitute for PI(4,5)P2 in EN2 secretion (Amblard et al., 2020a). This might reflect the fact that EN2 interaction with PI(4,5)P2 also depends on the acyl part of the molecule as it is not observed with the polar head alone. The contribution of the hydrophobic part of the bilayer is further supported by the direct interaction of EN2, but not FGF2, with a cholesterol-enriched PC bilayer, in agreement with its preferential association with cholesterol-enriched membranes (Joliot et al., 1997). However, cholesterol incorporation in PI(4,5)P2-containing membranes increases the affinity for both proteins (Temmerman et al., 2008; Amblard et al., 2020a), and plasma membrane depletion of cholesterol by methyl-ß-cyclodextrin impairs their secretion (Amblard et al., 2020a). Such interplay between PI(4,5)P2 and cholesterol are also observed for other proteins, through the induction of lipid phase demixing (Wang et al., 2016), or through stabilization of fluid PI(4,5)P2 domain by reducing electrostatic repulsion (Jiang et al., 2014).

The distinctive feature of PI(4,5)P2 interaction with FGF2, Tat and EN2 is to promote their translocation across the plasma membrane, well beyond its mere recruitment role. Upon interaction with PI(4,5)P2, FGF2 (Steringer et al., 2012) and Tat (Zeitler et al., 2015) assemble into oligomers that create pore-forming structures, ultimately leading to secretion. Although not formally ruled-out, formation of pores or oligomers was not observed with EN2. On the other hand, the conformation of the homeodomain motif analyzed by NMR is significantly modified in presence of membrane mimetics and is characterized by the partial insertion of the monomer within the acyl chains (Carlier et al., 2015). Differences in the translocation mechanism of the three proteins would not be so surprising in the light of the additional translocation properties of homeoproteins, leading to their internalization.

One could note that PI(4,5)P2 or cholesterol depletion does not fully inhibit homeoprotein secretion and furthermore, that the TransRush assay used to identify the role of PI(4,5)P2 in the secretion of EN2 specifically targets plasma membrane translocation events as it only quantify the secretion of the protein hooked at the inner side of the plasma membrane. Alternative secretion pathways involving other cell compartments could not be excluded.

## Homeoprotein Secretion and Internalization, Two Faces of a Same Process

As mentioned earlier, internalization is the first unusual trafficking property identified in several homeoproteins which also relies on unconventional mechanisms (Sagan et al., 2013). Its persistence at low temperatures that precludes endocytosis events (Joliot AH. et al., 1991) and the non-vesicular distribution of the internalized protein (Joliot A. et al., 1991), both agree with a translocation-driven process, in a way opposite to secretion. Contrasting with FGF2, extracellular delivery of recombinant EN2 quickly leads to its cytosolic accumulation that could also be quantified using the split-Nanoluciferase assay (Figures 1C,D).

**FIGURE 2:**
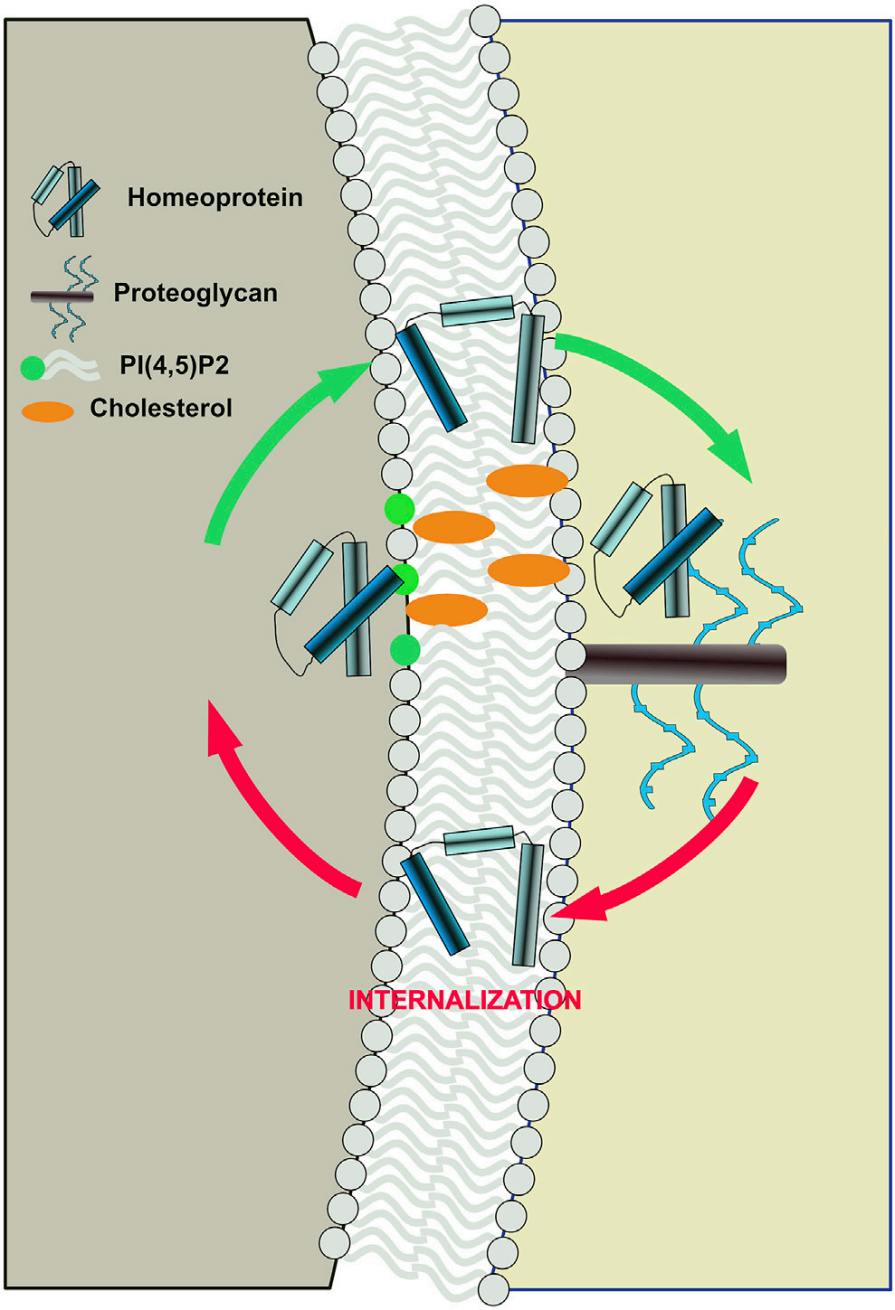
Proposed model of bidirectional translocation of EN2 homeoprotein across the plasma membrane. Interaction with PI(4,5)P2 and cholesterol at the inner side and with glycosaminoglycan at the outer side, act as a conformational switches for EN2 allowing its exchange between the polar and apolar environments.

Despite plasma membrane asymmetry, homeoprotein internalization surprisingly displays the same requirement for PI(4,5)P2 and cholesterol (Amblard et al., 2020a). Because of the strategic localization of the lipid polar heads at the interface between the polar and apolar environments constituted by the cytosol and the acyl chains respectively, PI(4,5)P2 interaction might act as a conformational switch for homeoproteins to exchange between the two environments in either direction (Figure 2). This implies that on the external face of the plasma membrane, devoid of PI(4,5)P2, other components would regulate these exchanges. Cell surface glycosaminoglycans (GAGs) are attractive candidates because they are critical for homeoprotein internalization (Beurdeley et al., 2012). Interestingly, homeoprotein interaction with GAGs appear to differ between various homeoproteins. Such specificity is illustrated by the binding of OTX2 to highly sulfated chondroitin (CS-E) at the surface of their target cells (Beurdeley et al., 2012), whereas EN2 preferentially interact with heparan sulfate (Figure 2) (Cardon et al., 2021). Interestingly, the presence of cell surface heparan sulfates at the outer leaflet of the plasma membrane is also mandatory for the completion of FGF2 secretion (Zehe et al., 2006).

## Homeoprotein Secretion and Sequence Requirement

EN2 homeoprotein was chosen to unravel the mechanism of homeoprotein secretion but most if not all homeoproteins are also able of intercellular transfer (Lee et al., 2019). Indeed, the homeodomain motif that defines the homeoprotein family is on its own sufficient to recapitulate the whole secretion/internalization process (Tassetto et al., 2005). Interestingly, the homeodomain is one of the most frequent motif retrieved in an unbiased PI(4,5)P2 interaction screen of a human protein fragment library (Bidlingmaier et al., 2011). The homeodomain belongs to the helix-loop-helix class of DNA-binding motifs and among the three alpha helices that compose its structure in solution, the third one is enriched in basic and aromatic residues and is critical for translocation (Derossi et al., 1994). This 16 amino-acid long motif, also known as penetratin, is a founder member of the cell-penetrating peptide family, used as vectors to deliver cytosolic cargoes into the cell (Kurrikoff et al., 2016). A second motif adjacent to the third helix is specifically required for secretion (Dupont et al., 2007). Since this motif also promotes nuclear export, its function is more likely linked to the trafficking of the protein towards the plasma membrane rather than to the translocation process *per se*.

Contrasting with FGF2 and Tat proteins, the residues within the homeodomain which are required for PI(4,5)P2 interaction remain uncharacterized but specific mutations in EN2 lying close to the homeodomain were shown to lower simultaneously the affinity for PI(4,5)P2 and the efficacy of transfer (secretion and internalization), possibly by impacting on homeodomain conformation or accessibility. The first one contains a substitution of two tryptophan residues with lysins in a motif known to mediate protein-protein interaction (Maizel et al., 1999). In the second one, a cysteine residue that promote EN2 homodimerization is substituted for a serine (Amblard et al., 2020b).

## Regulation of Homeoprotein Secretion

Unconventional and conventional secretion pathways differ not only by their mechanism but also how they might be regulated. Proteins that use unconventional pathways can localize in different parts of the cell and thus, before secretion, must reach the plasma membrane. Homeoproteins mainly reside in the nucleus, but they can be actively exported toward the cytosol thanks to the presence of a nuclear export signal (Maizel et al., 1999). Strikingly, mutations (Tassetto et al., 2005) or post-translational modifications (Maizel et al., 2002) that lower homeoprotein nuclear targeting also impair its secretion, suggesting that homeoprotein secretion requires its passage through the nucleus.

Homeoprotein intercellular transfer implies that, once secreted, they are internalized by adjacent cells, but the latter internalization can antagonize secretion when occurring in the secreting cell. Accordingly, we recently demonstrated that these two processes, secretion and internalization, are inversely regulated by the cell redox state (Amblard et al., 2020b), further supporting the view that they are two opposite faces of a same process. Near-physiological modulation of H_2_O_2_ levels through ectopic expression of H2O2-producing or -degrading enzymes reveals that high and low H_2_O_2_ levels favors secretion and internalization, respectively. As most of the motifs shown to regulate the transfer of EN2 reside outside the homeodomain, it is likely that the regulation of secretion would differ depending on the nature of the homeoprotein.

At the plasma membrane, PI(4,5)P2 and cholesterol levels are determinant to modulate the recruitment and subsequent secretion of homeoproteins, without excluding the implication of other partners as reported for FGF2 (Zacherl et al., 2015). The concentration and distribution of these two lipids can be regulated at multiple levels. Even within a single cell, plasma membrane PI(4,5)P2 levels can vary significantly, due for instance to the asymmetric distribution of the enzymes of that control their metabolism (Myeong et al., 2021). Whether secretion is polarized within a single cell is an open question. In particular, the possible involvement of cytonemes (Ramírez-Weber and Kornberg, 1999), key players in paracrine signaling by connecting producing and recipient cells, is an attractive hypothesis.

Although key players have been identified, a full understanding of the translocation mechanism of homeoprotein is still lacking. Interestingly, the minimal internalization sequence penetratin is able to induce lipid hexagonal phase when incubated with cellular lipid extracts (Berlose et al., 1996) and to induce lipid curvature in artificial vesicles (Lamazière et al., 2008), suggesting that induction of lipid bilayer remodeling might be part of the process.

## Physiology of Homeoprotein Secretion: Gate to Their Transfer

**FIGURE 3:**
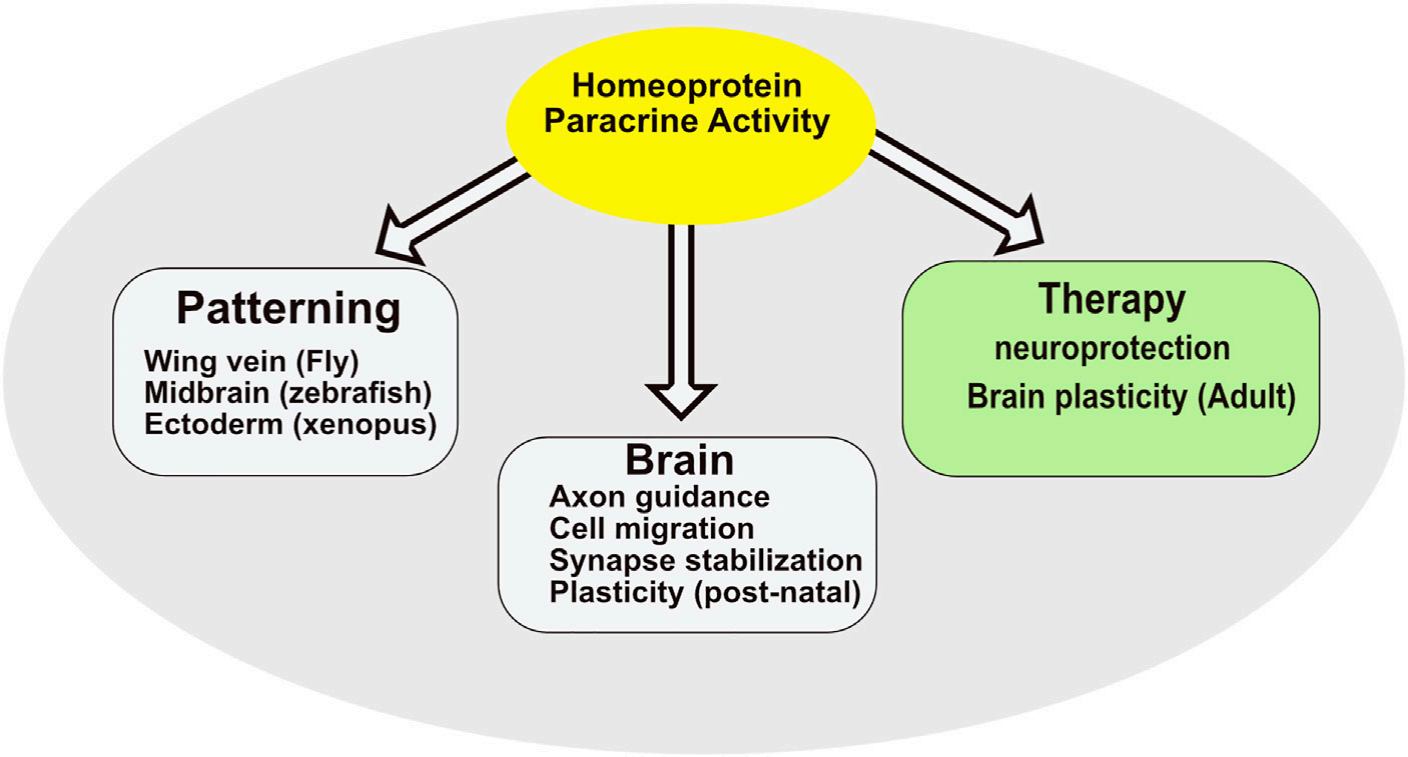
Summary of homeoprotein paracrine activity.

Visualization of homeoprotein secretion *in vivo* is hampered by the low endogenous levels of homeoproteins combined to their predominant nuclear localization but was reported in a few situations (Wizenmann et al., 2009; Kim et al., 2014). This contrasts with the multiple physiological functions requiring homeoprotein secretion (Figure 3). Until now, all these functions were linked to the transfer of the homeoprotein into recipient cells rather than to its extracellular presentation, for example by cytonemes, to classical receptors, yet to be identified. Even at the functional level, internalization and secretion are inseparable. The fact that both rely on similar mechanisms would explain how they have been co-opted simultaneously during evolution.

Transfer has been studied for handful of homeoproteins, with a focus on the physiological functions associated with this novel signaling pathway. Here we will restrict the discussion to animal cells, even though intercellular transfer was also reported in plants. This choice is dictated by the fact that, in plants, HP Knotted-1 transfer primarily involves plasmodesmata described as intercellular bridges (Ruiz-Medrano et al., 2004). Consequently, it may not correspond to a true secretion-internalization sequence although it displays unexpected similarities with the situation in animals (Tassetto et al., 2005).

## Early and Late Developmental Functions of Homeoprotein Transfer

Early in development, it was demonstrated that, in the *Drosophila* wing disk, transfer of Engrailed from the Patched domains towards anterior cells not expressing Engrailed is necessary for the formation of the anterior cross vein (Layalle et al., 2011). This was established by the extracellular expression of single chain antibodies (EN1/2-scFv) with Engrailed neutralizing activity. Interestingly, this induction requires a physiological synergic interaction with decapentaplegic (DPP) a morphogen of the TGFß family. As will be described below, such a signaling interaction between a HP and a classical signaling pathway was also reported in the chick tectum for axon guidance by EN2 and in the neural tube for the regulation of oligodendrocyte precursor (OPC) migration by PAX6 (Wizenmann et al., 2009; Di Lullo et al., 2011). Early morphogenetic activity of HP transfer is also involved in the patterning of the chick tectum by EN2 and the regulation of Cajal-Retzius cell migration by PAX6 in the mouse neuroepithelium (Rampon et al., 2015; Amblard et al., 2020b; Kaddour et al., 2020).

As already alluded to, still during development, but at slightly later stages, PAX6 transfer interacts with netrin to regulate OPC migration in the chick neural tube (Di Lullo et al., 2011). Still during late embryonic development, EN2 (possibly EN1), secreted by the chick optic tectum, where EN1 and EN2 show graded (anterior low, posterior high) expression, guides the migration of retinal ganglion cell (RGC) axons and participates in the distribution of nasal and temporal axons onto posterior and anterior tectum domains, respectively(Wizenmann et al., 2009). The latter paracrine EN2 activity was demonstrated *in vivo* thanks to the extracellular expression of neutralizing EN1/2-scFvs. *In vitro* studies allowed for the demonstration that the latter paracrine activity of EN2 requires its ability to regulate local protein translation, within growth cones, through the activation of eIF4I translation initiation factor. Further *in vitro* experiments led to conclude that the ability of EN2 to provoke the collapse of anterior RGC axon growth cones (Brunet et al., 2005), a guidance mechanism, requires a physiological interaction with EphrinA5 and Adenosine signaling at the growth cone level (Stettler et al., 2012). Similar results were reported for VAX1, the secretion of which at the optic chiasma is necessary for proper decussation (Kim et al., 2014). VAX1 activity follows its recognition of target optic chiasma cells through its binding to specific glycosaminoglycans.

## Post-Natal Physio-Pathological Functions of HP Homeoprotein Transfer

At post-natal stages the cerebral cortex adapts to the environment through morphological and physiological changes that take place during transient periods of plasticity, called critical periods (CPs) (Hensch, 2005). Such periods correspond to the maturation of a specific class of GABAergic interneurons expression parvalbumin (PV cells). These inhibitory interneurons form synapses with the cell body of excitatory pyramidal cells in layers III/IV of the cerebral cortex and their maturation during CP shifts the Excitatory/Inhibitory (E/I) balance toward inhibition (Hensch et al., 1998). A classic case of CP is the maturation of the visual cortex and is illustrated by the loss of visual acuity of an eye sutured during CP. This amblyopic phenotype can be reversed if the eye is reopened before the end of CP, but not after CP closure, unless adult plasticity is activated, in particular by blocking OTX2 import into PV cells (Beurdeley et al., 2012; Bernard et al., 2016).

It was shown that the opening of plasticity is triggered by the internalization of OTX2, a homeoprotein synthesized by the choroid-plexus and secreted into the cerebrospinal fluid (Sugiyama et al., 2008). The capture of OTX2 specifically by PV cells is permitted by the assembly of matrixial perineural nets (PNNs) that enwrap these cells and the specific binding of OTX2 to chondroitin sulfate (CSE/CSD) glycosaminoglycans (GAGs) present in the matrix. In the visual system, PNN assembly is induced by eye opening and photoreceptor activation (Sugiyama et al., 2008; Beurdeley et al., 2012; Miyata et al., 2012). The binding of OTX2 to CSE/CSDE GAGs is permitted by a small sequence, the RK peptide. Glycosaminoglycan-binding sequences similar to this RK peptide are present in a large number of other homeoproteins, including VAX1, EN1 and EN2 where they also trigger specific GAG recognition (Kim et al., 2014; Prochiantz and Di Nardo, 2015). Functional studies are only provided for these few proteins, but this observation suggests the existence of a sugar code for the specific recognition of target cells in homeoprotein transduction.

OTX2 is transported from the choroid plexus to PV cells throughout the cerebral cortex. Accordingly, its ability to regulate plasticity during development and in the adult is probably not limited to the visual system and was experimentally extended to the auditory cortex and the medial prefrontal cortex (Lee et al., 2017). A striking observation is that *Otx2* heterozygote mice are hypoanxious and that this trait is maintained in the adult, unless OTX2 is virally overexpressed in the choroid plexus (Vincent et al., 2021). Conversely, a normally anxious wild-type adult mouse can be made hypoanxious through the induction of an OTX2-scFv and the ensuing neutralization of OTX2 in the cerebrospinal fluid (Vincent et al., 2021).

## Other Example of Therapeutic Strategies Based on Homeoprotein Transduction

The possibility to cure experimental amblyopia or to regulate anxiety-like behaviors by modifying, permanently or transiently, OTX2 import by PV cells suggests that this novel signaling pathway might open new avenues in the study, possibly cure, of disease affecting the nervous system. Still for OTX2, its transfer within the retina from producing cells, probably bipolar neurons, to RGCs was demonstrated (Sugiyama et al., 2008) and from the retinal pigmented epithelium (RPE) to photoreceptors strongly suggested (Pensieri et al., 2021). The neutralization of extracellular OTX2 by an OTX2-scFv secreted by retinal parvalbumin producing cells leads to a decrease of visual acuity associated with the alteration of inner retinal functions and *Otx2* knock out, specifically in the RPE, leads to photoreceptor cell death (Torero Ibad et al., 2020; Pensieri et al., 2021). This putative trophic OTX2 activity was verified in the glaucoma model of induced excitotoxicity in the retina rapidly followed by RGC degeneration (Torero Ibad et al., 2011). OTX2 injection in the optic cup followed by its capture by RGCs completely protects against excitotoxicity and preserves visual acuity. In parallel it was shown that OTX2 promotes the survival of adult purified rodent RGCs *in vitro* and induces the regeneration of their axons from cultured retinal explants. This ability to promote axon regeneration was confirmed *in vivo* in the optic nerve crush paradigm (Ibad et al., 2022).

The potential therapeutic activity of EN1/2 proteins in the mouse was evaluated for mesencephalic dopaminergic (mDA) neurons of the Substantia Nigra pars compacta (SNpc) and for α-Motoneurons (αMNs) from the spinal cord ventral horns. EN1/2 expressed in the mDA neurons from the SNpc and Ventral Tegmental Area (VTA) mDA neurons exerts pro-survival activity in these cells as demonstrated by their progressive retrograde degeneration in the *En1* heterozygote mouse (*En1*-Het), associated with Parkinson-Disease (PD)-like motor and non-motor phenotypes (Sonnier et al., 2007; Nordströma et al., 2015). The bulk injection of EN1 or EN2 at the SNpc level, followed by its neuronal capture preserves mDA neurons from death in mouse and macaque PD models (Alvarez-Fischer et al., 2011; Rekaik et al., 2015; Blaudin de Thé et al., 2018; Thomasson, 2019). In the mouse, it was observed that EN1/2 acts at different levels, including an increase in the translation of mitochondrial complex-I, NdufS1 and NdufS3 proteins, a direct repression of genetic mobile elements of the LINE-1 family and the restoration of a healthy pattern for several heterochromatin marks, including MeCP2, Nucleolin, H3K27me3 and H3K9me3 (Alvarez-Fischer et al., 2011; Rekaik et al., 2015; Blaudin de Thé et al., 2018). This epigenetic activity probably explains why a single injection has long-lasting curative effects in mouse and macaque PD models.

α-Motoneurons in the ventral spinal cord do not express EN1/2 but are in post-synaptic contact with *En1*-expressing V1 interneurons and exhibit slow retrograde degeneration in the *En1*-Het mouse (Abonce et al., 2020). This degeneration is also observed *in vivo* following the viral expression of an EN1/2 scFv, demonstrating the EN1 secreted by V1 interneurons exerts a trophic activity on αMNs (Abonce et al., 2020). This trophic activity was confirmed by αMN protection by a single intrathecal injection of EN1 at the lumbar 5 (L5) level. The latter injection of EN1 is followed by its specific addressing to αMNs thanks to the EN1 GAG-binding domain. As shown for the mDA neurons, EN1 protecting activity is long-lasting with a duration of 2 months at least following a single injection, suggesting epigenetic mechanisms not yet studied in detail.

## Conclusion

In the light of their distinct cell trafficking requirement, how and when intracrine and paracrine activities of homeoproteins have been acquired along evolution is an intriguing question. Since transcriptional activity and intercellular transfer are both intimately linked to the presence of the homeodomain, this duality of activities might have been intrinsic to the advent of homeoproteins. Although displaying some similarities with prokaryote transcription factors of the helix-turn-helix class, genuine homeoproteins are first detected in unicellular eukaryotes (Derelle et al., 2007) and one of the most ancestral functions attributed to these proteins is linked to sexual mating (Sun et al., 2019) and consequently to intercellular communication. Thanks to their unique and unconventional secretion and internalization properties, homeoproteins might have constituted a primitive from of signaling, which does not require the presence of specific protein receptors. While being primitive, this mode of signaling has been conserved judging by the ability of most homeoproteins to transfer between cells, may be thanks to their ability to synergize with more stringent signaling pathways based on ligand/receptor interactions.
